# An epidemiological analysis of maxillofacial fractures: a 10-year cross-sectional cohort retrospective study of 1007 patients

**DOI:** 10.1186/s12903-021-01503-5

**Published:** 2021-03-17

**Authors:** Mihai Juncar, Paul Andrei Tent, Raluca Iulia Juncar, Antonia Harangus, Rivis Mircea

**Affiliations:** 1 Department of Oral and Maxillofacial Surgery, University of Oradea, Romania, Str. Piața 1 Decembrie, no.10, 410073 Oradea, Romania; 2 Research Center for Functional Genomics, Biomedicine and Translational Medicine, Iuliu Hatieganu” University of Medicine and Pharmacy, 400337 Cluj-Napoca, Romania; 3 Discipline of Oral Surgery, 2nd Department of Dental Medicine, “Victor Babeş” University of Medicine and Pharmacy, 2 Eftimie Murgu Sq., 300041 Timisoara, Romania

**Keywords:** Maxillofacial fractures, Maxillofacial trauma, Etiology, Epidemiology, Head and neck fractures

## Abstract

**Background:**

Epidemiological data is providing vital indicators for organizing the financial resources related to a particular type of trauma, estimating expenses and training of dental practioners and ambulatory medical staff for collaboration with a certain pattern of patients. Knowing the etiology and epidemiology of a certain pathology is significant for approaching its means of prevention.

**Methods:**

A 10-year retrospective statistical analysis of 1007 patients with maxillofacial fractures treated in a University Clinic of Oral and Maxillofacial Surgery in Romania was performed. The data were extracted from patients’ medical records. Statistical analysis was performed. A value of *p* < 0.05 was considered statistically significant.

**Results:**

The incidence of maxillofacial fractures was high among patients in the 20–29 age group (35.9%). Male patients (90.57%, M:F = 9.6:1), having a low level of education (46.60%) and living in urban areas (53.50%) were more affected. The main cause of maxillofacial fractures was interpersonal violence (59.37%), both in the mandibular and midface topographic regions (*p* = 0.001, *p* = 0.002). In urban areas, fractures caused by interpersonal violence and road traffic accidents were predominant, while in rural areas, most of the fractures were due to interpersonal violence, domestic accidents, work accidents and animal attacks (*p* = 0.001).

**Conclusions:**

Interpersonal violence is the main cause of maxillofacial fractures having epidemic proportions. Male patients aged 20–29 years with a low level of education represent the major risk category. Considering the wide area of interpersonal aggression, both the medical staff in the hospital and in the dental offices must be educated in order to collaborate with possible violent patients. Dentists must be prepared to work on a post-traumatic dento-periodontal field. Taking all measures to prevent inter-human aggression is imperative and will lead to a major decrease in maxillofacial fractures and an overall increase of oral health in a population.

**Supplementary Information:**

The online version contains supplementary material available at 10.1186/s12903-021-01503-5.

## Background

Facial trauma is continually increasing worldwide, being the most frequent type of pathology diagnosed and treated in oral and maxillofacial surgery services [1, 2]. The severity of maxillofacial trauma varies depending on the type of etiology, the kinetic energy of the wounding agent and nevertheless on the dynamics between the wounding agent and the recipient [3, 4]. Injuries can be present both isolated or as part of a polytrauma, coexisting with intracranial, cerebral, ocular, spinal, thoracic or abdominal lesions that can significantly increase the complexity and morbidity of the case [5, 6]. Alteration of the facial features of an individual may have functional, psychological, social and, not least, professional consequences, difficult to reverse over time [7, 8]. In this context, the management of maxillofacial fractures can be complex, involving a multidisciplinary approach and high costs [1–3].

Maxillofacial fractures have a direct impact on oral health, opening of the fracture site in the oral cavity may favor the appearance of infections or osteitis [1–4]. Also associated dento-periodontal trauma can range from simple coronary fractures to dental avulsions followed by edentations, that may imply a secondary complex oral rehabilitation treatment which involves high costs [5, 6]. The implications of a mandibulo-maxilary fixation (MMF) treatment should not be neglected either. The MMF is accompanied by difficulties in maintaining proper oral hygiene and periodontal distress [7]. Therefore the prevention of maxillo-facial fractures will lead directly to an overall increase in the public oral health [1–7].

The causes of maxillofacial fractures and the category of affected patients differ significantly depending on the socioeconomic, cultural, religious, educational and demographic status [9, 10]. Determining the etiological and epidemiological factors of a disease in a certain geographical area, provides extremely important data for implementing adequate prevention, diagnostic and treatment methods [7–10]. For this purpose, many studies have been conducted worldwide, but no consensus has yet been reached regarding the main etiology of maxillofacial fractures, because of great differences from one region to another and evolution in time [5–12]. In our geographical region, there are currently no studies related to the etiology of the entire facial skeleton fractures [3, 13]. In this context, we consider this shortcoming a public health emergency.

The aim of this study is to determine the epidemiology and the main etiology of maxillofacial fractures, as well as to correlate them in order to identify the main categories of affected patients depending on traumatic etiology. The results of this research will be useful in implementing legislative norms for the prevention of maxillofacial fractures, increasing general oral health, as well as in training the medical staff and dentists for the adequate management of this pathology and collaboration with a certain type of patients.

## Methods

This study was conducted in a tertiary center of oral and maxillo-facial surgery from Romania. The patients were selected retrospectively over a 10-year period. We mention that the addressability of maxillofacial trauma in the host center of the study comes from a wide geographical area in Eastern Europe. All patients included in the study signed an informed consent at the time of their admission to the clinical service, by which they agreed to the use of their anonymized medical data for scientific research purposes. In the case of patients under the age of 18, the informed consent was signed by the parent or their legal guardian. The study was approved by the Ethics Committee of Oradea University (IRB No. 35698/19.02.2018) and was therefore performed in accordance with the ethical standards laid down in the 2008 Declaration of Helsinki and its later amendments.

The study inclusion criteria were the following: presence of at least one fracture line in the facial skeleton, an episode of acute trauma in the disease history, paraclinical examinations (radiographic or computed tomographic examination) confirming the clinical diagnosis of fracture and evidencing its location and characteristics, treatment of the fracture performed in the study host institution. We mention that because of the epidemiological nature of this study, the postoperative follow-up period of the patients did not represent an inclusion or exclusion criteria of the participants.

The criteria of exclusion from the study were: patient without any fracture lines in the facial skeleton, pathological bone fracture, absence of complementary imaging investigations, treatment performed in another service. All patients with incomplete data in the medical record sheets were excluded from this study.

The data were extracted from patients’ medical records, and the following variables divided into subgroups were monitored: sex (male/female), age (divided into 10-year age groups), environment of origin (urban/rural), level of education (no education—patients not having completed the 1st grade of primary school, primary school—the highest education level completed, middle school—the highest education level completed, high school—the highest education level completed, university studies—patients having graduated from a faculty), traumatic etiology, location of fracture lines in the facial skeleton (mandible/midface/combined), location of fracture lines in the mandible (symphyseal/parasymphyseal/lateral/angle/ramus/condylar process (head or intracapsular, subcondylar region)/coronoid process/alveolar process), location of fracture lines in the midface (Le Fort I/Le Fort II/Le Fort III/ zygomatic complex/nasal bones/alveolar margin/orbit/anterior maxillary sinus wall). We mention that in the subcondylar region category we included both patients with condylar neck and subcondylar fractures.

To prevent bias all observation sheets were checked twice by both the author who collected the data and a member of the statistical team.

The size of the study was due to the period of time in which the data were collected, namely 10 years.

The data extracted from the medical sheets of the patients were centralized electronically by the authors using Microsoft Excel Software. Initially, descriptive statistics of the collected data was performed with an accuracy of two decimal percentage. For performing the statistical analysis and the statistical correlations between variables, MedCalc Statistical Software version 19.2 (MedCalc Software bvba, Ostend, Belgium;53 https://www.medcalc.org; 2020) was used. Thus, nominal information was expressed in the statistical analysis as percentage and frequency. Using the chi-square test, the frequencies of a nominal variable between the categories of another nominal variable were compared. For a result to be considered statistically significant a value of *p* < 0.05 was necessary.

## Results

1569 clinical sheets with maxillo-facial trauma were found in our hospital’s archive in the 10 year chosen timeline for this study. 562 patients were excluded from this study as follows: 251 trauma patients had only facial contusions without any fracture line being identifiable, 121 patients had incomplete data regarding the environment of origin, 175 patients had incomplete data regarding the level of education and 15 patients did not report the cause of trauma. 1007 patients with a total number of 1661 fracture lines in the facial skeleton were included in this study.

The most affected age group was 20–29 years n = 360 (35.90%), followed by 30–39 years n = 182 (18.30%), 10–19 years n = 165 (16.40%), 40–49 n = 124 (12.30%), 50–59 n = 92 (9.10%), 60–69 n = 44 (4.40%), 70–79 n = 28 (2.40%) and 0–9 years n = 12 (1.20%).

The majority of the patients were male, n = 912 (90.60%), women representing a small proportion, n = 95 (9.40%). The M/F ratio = 9.6/1.

Patients living in urban areas, n = 539 (53.50%), were more affected than those living in rural areas, n = 456 (46.50%).

Patients with no education, n = 477 (46.40%), had the highest frequency of maxillofacial fractures, being followed by patients having middle school studies, n = 218 (22.00%), high school studies, n = 175 (17.60%), university studies, n = 71 (7.20%), and primary school studies n = 66 (6.70%).

The main traumatic etiology was interpersonal violence (IPV), n = 598 (59.38%), followed by falls, n = 162 (16.02%), road traffic accidents (RTA), n = 85 (8.41%), domestic accidents, n = 59 (5.84%), animal attacks, n = 56 (5.54%), sports injuries, n = 36 (3.56%), and work accidents, n = 15 (1.48%).

The type of traumatic etiology was correlated with patients’ age group (Table 1) and sex (Table 2). Children under the age of 9 years and patients aged over 70 years more frequently suffered maxillofacial fractures from falling, while patients aged between 10 and 69 years had more fractures caused by interpersonal violence. This result was statistically significant (*p* = 0.004). The incidence of men with maxillofacial fractures was high in all categories of traumatic etiology, the result being statistically significant (*p* = 0.003).

**Table 1:** Distribution of the types of traumatic etiology depending on age

	Etiology of trauma							Total
	IPV	RTA	Domestic accident	Sports injury	Work accident	Fall	Animal attack	
Age								
0–9	1	2	1	0	0	5	3	12
	0.2%	2.4%	1.7%	0.0%	0.0%	3.2%	5.4%	1.2%
10–19	98	17	3	11	0	24	12	165
	16.5%	20.0%	5.1%	30.6%	0.0%	15.2%	21.4%	16.4%
20–29	261	30	19	12	4	28	6	360
	43.9%	35.3%	32.2%	33.3%	26.7%	17.7%	10.7%	35.9%
30–39	100	14	15	10	3	30	10	182
	16.8%	16.5%	25.4%	27.8%	20.0%	19.0%	17.9%	18.3%
40–49	64	11	7	1	5	29	7	124
	10.8%	12.9%	11.9%	2.8%	33.3%	18.4%	12.5%	12.3%
50–59	48	6	7	2	2	19	8	92
	8.1%	7.1%	11.9%	5.6%	13.3%	12.0%	14.3%	9.1%
60–69	16	3	4	0	1	11	9	44
	2.7%	3.5%	6.8%	0.0%	6.7%	7.0%	16.1%	4.4%
> 70	6	2	3	0	0	16	1	28
	1.0%	2.4%	5.1%	0.0%	0.0%	7.6%	1.8%	2.4%
Total *P* = 0.004	598	85	59	36	15	162	56	1007
	100.0%	100.0%	100.0%	100.0%	100.0%	100.0%	100.0%	100.0%

*IPV* interpersonal violence, *RTA* road traffic accident

**Table 2:** Distribution of the types of traumatic etiology depending on sex

	Etiology of trauma							Total
	IPV	RTA	Domestic accident	Sports injury	Work accident	Fall	Animal attack	
Sex								
F	35	25	2	0	1	25	7	95
	5.9%	29.4%	3.4%	0.0%	6.7%	15.8%	12.5%	9.4%
M	559	60	57	36	14	137	49	912
	94.1%	70.6%	96.6%	100.0%	93.3%	84.2%	87.5%	90.6%
Total *P* = 0.003	598	85	59	36	15	162	56	1007
	100.0%	100.0%	100.0%	100.0%	100.0%	100.0%	100.0%	100.0%

*IPV* interpersonal violence, *RTA* road traffic accident

The type of traumatic etiology was correlated with patients’ level of education and environment of origin (Table 3). In urban areas, maxillofacial fractures caused by aggression and road traffic accidents were predominant, while in rural areas, those caused by animal attacks and domestic accidents (*p* = 0.001). Regarding the level of education, interpersonal violence was the main etiology in all categories. These results were statistically significant (*p* = 0.005).

**Table 3:** Distribution of the types of traumatic etiology depending on the level of education and environment

Level of education	Etiology of trauma							
	IPV	RTA	Domestic accident	Sports injury	Work accident	Fall	Animal attack	Total
No education	276	36	27	10	9	85	34	477
	45.2%	41.7%	44.8%	25.7%	60.0%	52.6%	60.0%	46.4%
Primary school	32	4	0	1	1	15	13	66
	5.1%	4.8%	0.0%	2.9%	6.7%	9.7%	23.6%	6.7%
Middle school	135	14	21	7	3	31	7	218
	23.1%	16.7%	36.2%	20.0%	20.0%	20.1%	12.7%	22.0%
High school	114	21	8	9	1	20	2	175
	19.3%	25.0%	13.8%	25.7%	6.7%	13.0%	3.6%	17.6%
University studies	41	10	3	9	1	7	0	71
	7.0%	11.9%	5.2%	25.7%	6.7%	4.5%	0.0%	7.2%
Total *P* = 0.005	598	85	59	36	15	158	56	1007
	100.0%	100.0%	100.0%	100.0%	100.0%	100.0%	100.0%	100.0%
Environment								
R	263	35	33	9	9	72	47	468
	43.8%	41.2%	55.9%	25.0%	60.0%	45.6%	83.9%	46.5%
U	335	50	26	27	6	86	9	539
	56.2%	58.8%	44.1%	75.0%	40.0%	54.4%	16.1%	53.5%
Total *P* = 0.001	598	85	59	36	15	158	56	1007
	100.0%	100.0%	100.0%	100.0%	100.0%	100.0%	100.0%	100.0%

*IPV* interpersonal violence, *RTA* road traffic accident

Of all patients, 629 (62.46%) had strictly mandibular fractures with a total of 1099 fracture lines, 297 (29.49%) had strictly midface fractures, and 81 (8.04%) had concomitant mandibular and midface fractures. The most frequent fracture site in the mandible was the angle 28.84% (n = 317), followed body 24.29% (n = 276), subcondylar region 22.02% (n = 242), parasymphyseal 17.38% (n = 191), symphyseal 3.18% (n = 35), ramus 2.00% (n = 22), coronoid process 1.18% (n = 13) and alveolar process 1.09% (n = 12). No intracapsular condylar fractures were identified. Mandibular fractures were most frequently caused by interpersonal violence *p* = 0.001. Table 4 shows the distribution of the fracture lines location depending on etiology. Similarly to the situation of lower face fractures, interpersonal violence was the main cause of midface fractures. In Table 5, the distribution of the fracture lines depending on location and cause can be observed (Additional files 1, 2).

**Table 4:** Distribution of mandibular fractures depending on etiology

	Etiology of trauma							Total
	IPV	RTA	Domestic accident	Sports injury	Work accident	Fall	Animal attack	
Mandibular fractures								
Absent	140	37	30	20	6	49	19	301
	23.6%	43.5%	50.8%	55.6%	40.0%	31.0%	33.9%	29.9%
Symphyseal	3	3	0	0	1	1	0	8
	0.5%	3.5%	0.0%	0.0%	6.7%	0.6%	0.0%	0.8%
Parasymphyseal	18	3	4	0	0	7	2	34
	3.0%	3.5%	6.8%	0.0%	0.0%	4.4%	3.6%	3.4%
Body	37	7	2	2	2	15	6	71
	6.1%	8.2%	3.4%	5.6%	13.3%	9.5%	10.7%	7.1%
Angle	107	0	4	2	1	19	3	136
	17.8%	0.0%	6.8%	5.6%	6.7%	12.0%	5.4%	13.5%
Ramus	2	0	1	0	0	1	2	6
	0.3%	0.0%	1.7%	0.0%	0.0%	0.6%	3.6%	0.6%
Subcondylar region	39	4	3	3	0	16	4	69
	6.6%	4.7%	5.1%	8.3%	0.0%	10.1%	7.1%	6.9%
Coronoid process	0	2	1	1	0	1	0	5
	0.0%	2.4%	1.7%	2.8%	0.0%	0.6%	0.0%	0.5%
Alveolar process	3	1	0	1	0	1	2	8
	0.5%	1.2%	0.0%	2.8%	0.0%	0.6%	3.6%	0.8%
Multiple	249	28	14	7	5	48	18	369
	41.6%	32.9%	23.7%	19.4%	33.3%	30.4%	32.1%	36.6%
Total *P* = 0.001	598	85	59	36	15	158	59	1007
	100%	100%	100%	100%	100%	100%	100%	100%

*IPV* interpersonal violence, *RTA* road traffic accident

**Table 5:** Distribution of midface fractures depending on etiology

	Etiology of trauma							Total
	IPV	RTA	Domestic accident	Sports injury	Work acc	Fall	Animal attack	
Midface fractures								
Absent	428	25	27	15	7	95	29	626
	71.5%	29.4%	45.8%	41.7%	46.7%	60.1%	51.8%	62.2%
Le Fort I	0	0	1	0	0	2	1	4
	0.0%	0.0%	1.7%	0.0%	0.0%	1.3%	1.8%	0.4%
Le Fort II	5	1	0	0	1	2	0	9
	0.8%	1.2%	0.0%	0.0%	6.7%	1.3%	0.0%	0.9%
Le Fort III	1	1	2	0	1	0	0	5
	0.2%	1.2%	3.4%	0.0%	6.7%	0.0%	0.0%	0.5%
Zygomatic	88	19	10	11	2	29	13	172
	14.6%	22.4%	16.9%	30.6%	13.3%	18.4%	23.2%	17.1%
Nasal bones	30	6	4	7	0	10	0	57
	5.1%	7.1%	6.8%	19.4%	0.0%	6.3%	0.0%	5.7%
Alveolar process	9	6	4	1	0	4	3	27
	1.5%	7.1%	6.8%	2.8%	0.0%	2.5%	5.4%	2.7%
Orbit	0	1	2	1	0	1	0	5
	0.0%	1.2%	3.4%	2.8%	0.0%	0.6%	0.0%	0.5%
Anterior maxillary sinus wall	1	0	0	0	0	0	0	1
	0.2%	0.0%	0.0%	0.0%	0.0%	0.0%	0.0%	0.1%
Multiple	36	26	9	1	4	15	10	101
	6.1%	30.6%	15.3%	2.8%	26.7%	9.5%	17.9%	10.0%
Total *P* = 0.002	598	85	59	36	15	158	56	1007
	100%	100%	100%	100%	100%	100%	100%	100%

*IPV* interpersonal violence, *RTA* road traffic accident

## Discussion

This study evidences a high incidence of maxillofacial fractures in the 20–29 age group, which is in accordance with the results reported by other authors [2, 4, 9–20]. This finding can be due to the fact that during this life decade, individuals are more socially, professionally and physically active, being more exposed to trauma [2–4]. Young people are more extroverted and participate in social events more often [12]. In these circumstances, consumption of alcohol or recreational drugs predisposes them to interpersonal conflicts which can lead to physical aggression [12, 21, 22]. For the same reasons, the patients belonging to this life decade are predisposed to road traffic accidents due to their lack of experience, breaking of traffic rules or high-speed driving [15–23]. Contrary to our findings, in other studies the incidence of maxillofacial fractures is predominant in the 30–39 age group [24–26]. This can be attributed to global population aging [17].

Maxillofacial fractures predominate among men, both in this study and in the literature [9–23]. Behaviorally, men are more predisposed to engage in interpersonal conflicts than women, therefore the risk to suffer a fracture caused by aggression being higher [27]. With respect to daily activities, men are more frequently involved in physical work, for example in construction works, being more predisposed to work accidents [23–27]. Extreme sports or contact sports are also predominantly practiced by men, who are at a higher risk for maxillofacial fractures caused by sports injuries [2–6]. However, in developed countries, where women are involved in society as much as men, the male/female ratio tends to decrease [28–30].

A higher incidence of maxillofacial fractures was found in urban areas in our study, which is in accordance with the results of other publications [27, 31–33]. The high density of the population in the urban environment, the great discrepancies between social classes, the easy access to alcohol or narcotic substances are factors that contribute to increasing the risk of interpersonal conflicts [27, 31–33]. Also, the city infrastructure based on highways allowing high-speed circulation of vehicles, concomitantly with the multiplying number of vehicles, leads to an increase in the risk of road traffic accidents [27–33]. Contrary to our findings, other studies indicate a higher frequency of maxillofacial fractures in rural areas [34]. These discrepancies can be explained by the differences between the regions served by the institutions in which those studies were carried out [34]. In our study, an increased incidence of interpersonal violence in both environments was found. This result is uncommon and rarely found in the existing literature [2, 6]. Also our institution where the study took place serves many counties composed of both urban and rural regions. This fact can also explain our result.

In our study we found that most of the affected patients had a low level of education. This result is also reported by other authors [17, 35, 36] A lower education level predisposes to unemployment, low social status, material deficiencies and implicitly, limited access to healthcare services [17]. All these factors can lead to frustration and depression which, supported by alcohol or drug consumption, can lead to conflicts and interpersonal violence [22, 23, 35, 36]. These findings are upheld by other authors who certify the small number of traumas secondary to aggression in a population with a high education level [23, 36]. Also, in the context of the absence of an intellectual qualification, people are forced to earn their living by practicing unqualified physical work [17]. The risk to suffer a maxillofacial fracture through a work accident is higher in this context compared to the intellectual work environment [17]. According to our and other authors’ results, the increase in the education level of a population is a significant method for the prevention of maxillofacial fractures [22, 23, 36]. Although the highest incidence of fractures caused by interpersonal violence was found among patients without education, our study evidences the predominance of interpersonal violence as a main etiological factor in the other education level categories as well. This fact is rarely found in the literature and it must be considered an alarm signal in public health [22–27].

The most frequent mechanism of maxillofacial fractures was interpersonal violence, a result also found in studies conducted in other geographical areas such as Germany [37], Brazil [6, 33], USA [24, 31, 39], Italy [26, 38], Australia [7], Norway [2, 29] or Sweden [40]. The incidence of interpersonal violence has increased over the past decade in developed countries [29–39]. Recent European studies confirm a shift of the main etiological factor of maxillofacial fractures from road traffic accidents or sports injuries to interpersonal violence [2, 26, 40]. The cultural, social and educational mosaic in the cities of developed countries is an environment that constantly predisposes to interpersonal conflicts and implicitly, to maxillofacial fractures [26–39]. The interrelation between interpersonal violence and alcohol found in developed countries should not be overlooked either [37–40]. For example, in Arab countries where alcohol consumption is restricted or even forbidden by law, interpersonal violence has a low incidence [5, 41].

In contrast to our findings, in studies conducted in regions such as Nigeria [42], Uganda [20], India [17, 19, 27], Egypt [1], Saudi Arabia [5, 41], China [19], South Korea [15, 30], Malaysia [16, 43] or Iran [44], maxillofacial fractures caused by road traffic accidents are predominant. The high incidence of maxillofacial fractures through road traffic accidents in developing countries is due to many factors: poorly defined traffic rules, deliberately driving unapproved or uninspected vehicles and, not least, inadequate traffic lighting and marking of roads [5, 17–20, 41–44]. A high frequency of road traffic accidents is also reported in developed countries with an increased population density, where such accidents are caused by the carelessness and non-compliance of drivers with the traffic rules [19–30]. In our country, the well-defined traffic rules, as well as the high penalties for their infringement, have lately led to a considerable reduction in the number of maxillofacial fractures caused by road traffic accidents. In contrast to the above publications, other authors report falling as the main cause of maxillofacial fractures [28, 45–47]. This can be due to effective prevention of interpersonal violence and traffic accidents in the geographical areas where the studies were conducted [47]. Global population aging should not be overlooked either, as the predisposition of elderly persons to facial trauma from falling is well known [5, 40–47]. This is also evidenced by our findings.

Maxillofacial fractures caused by work accidents, domestic accidents or animal attacks had a low incidence in this study, being predominant in rural areas. These findings are consistent with those reported in the literature [1–25].

The mandible was the most fractured bone in this study, in accordance with the literature data [1–25] This finding is not surprising given the prominence of the mandible in the lower face, being directly exposed to trauma [1–25]. Regarding the most frequent location of the fracture line in the mandible, authors’ opinions diverge. According to our and other authors’ findings, mandibular angle fractures are the most frequent [47, 49], while other authors report the highest frequency of subcondylar region fractures [23, 50], or parasymphyseal mandibular fractures [52]. The location of the fracture line in the mandible varies depending on the type, texture, place of action, speed and kinetic energy of the wounding agent on the one hand, and on the position of the head and time of impact on the other hand [48–52]. This explains the discrepancies described in the literature related to this aspect [48–52].

In the midface, the most fractured bone was the zygomatic bone, which is supported by other authors [3–10, 17, 20, 27]. The zygomatic bone is the lateral pillar of the midface, absorbing most of the traumatic forces in this region [20, 27]. The fact that individuals tend to turn their head at the time of the impact in order to avoid frontal or ocular contact should also be considered [17–20, 27]. All this makes the zygomatic complex more susceptible to fracture [20, 27]. Contrary to our findings, other authors indicate the highest incidence of nasal bone fractures [42, 53] or orbital fractures [26, 34, 54]. The sagittal prominence of the nasal bones in the face explains the high incidence of fractures at this level [42, 53]. Biomechanically, the nasal bones have a decreased resistance to trauma [42, 53, 54]. The fact that in this study lateral orbital wall and orbital floor fractures were included as zygomatic complex fractures category, explains the small number of orbital fractures in our findings. In our study the majority of patients with multiple fracture lines in the midface caused by interpersonal violence is higher than those caused by RTAs. This is uncommon and rarely reported in the literature [1, 18, 24, 25]. Patients suffer multiple fracture lines in the midface usually secondary to RTAs due to the high kinetic energy developed, rather than human aggression [1, 18, 24, 25].

We believe that this study is providing vital information regarding the etiology and epidemiology of maxillo-facial fractures. This information can be used for managing the distribution of financial resources in healthcare services, preparing the doctors and nurses in order to relate to a certain type of the patients, and not least it can be used for implementing preventive measures regarding this particular pathology.

However there are several limitations that have to be taken into consideration regarding this study. A major limitation is that this study is a retrospective one. In these circumstances, the data taken from the observation sheets may be incomplete or erroneously recorded at the time of patient submission. A randomised controlled trial study should be done in the future to avoid these shortcomings. The possibility of people intentionally misreporting the cause of the trauma must also be considered. This occurs frequently in the case of inter-personal aggression, victims often indicate a different cause of trauma out of fear or to avoid certain legal implications.

## Conclusions

Interpersonal violence represents the main etiology of maxillofacial fractures, having epidemic proportions in rural and urban areas. Male patients aged between 20 and 29 years with a low level of education represent the major risk category. The mandible is the most fractured bone of the face, followed by the zygomatic bone. Increasing the legal punishments in case of aggression, as well as the global increase of education in our population can lead to an overall decrease of the number of maxillofacial fractures. Measures for educating the medical and auxiliary staff to communicate with aggressive patients are imperative in order to facilitate the speed of diagnosis and implementation of emergency treatment.

## Supplementary Information

**Additional file 1.** Patient data - 1007 patients with codifications.

**Additional file 2.** Initial statistical analysis.

## Data Availability

The datasets generated during and analysed during the current study are not publicly available due to absence of a public storage online platform at our university, but are available from the corresponding author on reasonable request.
