# Supplementary material for: An epidemiological analysis of maxillofacial fractures: a 10-year cross-sectional cohort retrospective study of 1007 patients
Source: BMC Oral Health. 2021 Mar 17;21:128. doi: 10.1186/s12903-021-01503-5 (PMC7968332; doi:10.1186/s12903-021-01503-5)
Supplement: Supplementary file 1 — Additional file 1. Patient data - 1007 patients with codifications. [file 12903_2021_1503_MOESM1_ESM.docx]

**Age and Etiology of Trauma**

| **Crosstab** | | | | | | | | | | | | |
| --- | --- | --- | --- | --- | --- | --- | --- | --- | --- | --- | --- | --- |
|  | | | Etiologia traumei | | | | | | | | | Total |
|  |  |  | .0 | 1.0 | 1.9 | 2.0 | 3.0 | 4.0 | 5.0 | 7.0 | 8.0 |  |
| Varsta | 0-9 | Count | 0 | 1 | 0 | 2 | 1 | 0 | 0 | 5 | 3 | 12 |
|  |  | % within Etiologia traumei | 0.0% | 0.2% | 0.0% | 2.4% | 1.7% | 0.0% | 0.0% | 3.2% | 5.4% | 1.2% |
|  | 10-19 | Count | 0 | 98 | 0 | 17 | 3 | 11 | 0 | 24 | 12 | 165 |
|  |  | % within Etiologia traumei | 0.0% | 16.5% | 0.0% | 20.0% | 5.1% | 30.6% | 0.0% | 15.2% | 21.4% | 16.4% |
|  | 20-29 | Count | 0 | 261 | 2 | 30 | 19 | 12 | 4 | 28 | 6 | 362 |
|  |  | % within Etiologia traumei | 0.0% | 43.9% | 100.0% | 35.3% | 32.2% | 33.3% | 26.7% | 17.7% | 10.7% | 35.9% |
|  | 30-39 | Count | 2 | 100 | 0 | 14 | 15 | 10 | 3 | 30 | 10 | 184 |
|  |  | % within Etiologia traumei | 100.0% | 16.8% | 0.0% | 16.5% | 25.4% | 27.8% | 20.0% | 19.0% | 17.9% | 18.3% |
|  | 40-49 | Count | 0 | 64 | 0 | 11 | 7 | 1 | 5 | 29 | 7 | 124 |
|  |  | % within Etiologia traumei | 0.0% | 10.8% | 0.0% | 12.9% | 11.9% | 2.8% | 33.3% | 18.4% | 12.5% | 12.3% |
|  | 50-59 | Count | 0 | 48 | 0 | 6 | 7 | 2 | 2 | 19 | 8 | 92 |
|  |  | % within Etiologia traumei | 0.0% | 8.1% | 0.0% | 7.1% | 11.9% | 5.6% | 13.3% | 12.0% | 14.3% | 9.1% |
|  | 60-69 | Count | 0 | 16 | 0 | 3 | 4 | 0 | 1 | 11 | 9 | 44 |
|  |  | % within Etiologia traumei | 0.0% | 2.7% | 0.0% | 3.5% | 6.8% | 0.0% | 6.7% | 7.0% | 16.1% | 4.4% |
|  | >70 | Count | 0 | 6 | 0 | 2 | 3 | 0 | 0 | 12 | 1 | 24 |
|  |  | % within Etiologia traumei | 0.0% | 1.0% | 0.0% | 2.4% | 5.1% | 0.0% | 0.0% | 7.6% | 1.8% | 2.4% |
| Total | | Count | 2 | 594 | 2 | 85 | 59 | 36 | 15 | 158 | 56 | 1007 |
|  |  | % within Etiologia traumei | 100.0% | 100.0% | 100.0% | 100.0% | 100.0% | 100.0% | 100.0% | 100.0% | 100.0% | 100.0% |

| **Chi-Square Tests** | | | |
| --- | --- | --- | --- |
|  | Value | df | Asymp. Sig. (2-sided) |
| Pearson Chi-Square | 157.626^a^ | 56 | .004 |
| Likelihood Ratio | 151.790 | 56 | .004 |
| Linear-by-Linear Association | 34.411 | 1 | .0003 |
| N of Valid Cases | 1007 |  |  |
| a. 39 cells (54.2%) have expected count less than 5. The minimum expected count is .02. | | | |

**Sex And Etiology of Trauma**

| **Crosstab** | | | | | | | | | | | | |
| --- | --- | --- | --- | --- | --- | --- | --- | --- | --- | --- | --- | --- |
|  | | | Etiologia traumei | | | | | | | | | Total |
|  |  |  | .0 | 1.0 | 1.9 | 2.0 | 3.0 | 4.0 | 5.0 | 7.0 | 8.0 |  |
| Sex | F | Count | 0 | 35 | 0 | 25 | 2 | 0 | 1 | 25 | 7 | 95 |
|  |  | % within Etiologia traumei | 0.0% | 5.9% | 0.0% | 29.4% | 3.4% | 0.0% | 6.7% | 15.8% | 12.5% | 9.4% |
|  | M | Count | 2 | 559 | 2 | 60 | 57 | 36 | 14 | 133 | 49 | 912 |
|  |  | % within Etiologia traumei | 100.0% | 94.1% | 100.0% | 70.6% | 96.6% | 100.0% | 93.3% | 84.2% | 87.5% | 90.6% |
| Total | | Count | 2 | 594 | 2 | 85 | 59 | 36 | 15 | 158 | 56 | 1007 |
|  |  | % within Etiologia traumei | 100.0% | 100.0% | 100.0% | 100.0% | 100.0% | 100.0% | 100.0% | 100.0% | 100.0% | 100.0% |

| **Chi-Square Tests** | | | |
| --- | --- | --- | --- |
|  | Value | df | Asymp. Sig. (2-sided) |
| Pearson Chi-Square | 63.415^a^ | 8 | .003 |
| Likelihood Ratio | 55.197 | 8 | .012 |
| N of Valid Cases | 1007 |  |  |
| a. 6 cells (33.3%) have expected count less than 5. The minimum expected count is .19. | | | |

**Mediu * Etiologia traumei**

| **Crosstab** | | | | | | | | | | | | |
| --- | --- | --- | --- | --- | --- | --- | --- | --- | --- | --- | --- | --- |
|  | | | Etiologia traumei | | | | | | | | | Total |
|  |  |  | .0 | 1.0 | 1.9 | 2.0 | 3.0 | 4.0 | 5.0 | 7.0 | 8.0 |  |
| Mediu | R | Count | 2 | 260 | 1 | 35 | 33 | 9 | 9 | 72 | 47 | 468 |
|  |  | % within Etiologia traumei | 100.0% | 43.8% | 50.0% | 41.2% | 55.9% | 25.0% | 60.0% | 45.6% | 83.9% | 46.5% |
|  | U | Count | 0 | 334 | 1 | 50 | 26 | 27 | 6 | 86 | 9 | 539 |
|  |  | % within Etiologia traumei | 0.0% | 56.2% | 50.0% | 58.8% | 44.1% | 75.0% | 40.0% | 54.4% | 16.1% | 53.5% |
| Total | | Count | 2 | 594 | 2 | 85 | 59 | 36 | 15 | 158 | 56 | 1007 |
|  |  | % within Etiologia traumei | 100.0% | 100.0% | 100.0% | 100.0% | 100.0% | 100.0% | 100.0% | 100.0% | 100.0% | 100.0% |

| **Chi-Square Tests** | | | |
| --- | --- | --- | --- |
|  | Value | df | Asymp. Sig. (2-sided) |
| Pearson Chi-Square | 46.548^a^ | 8 | .001 |
| Likelihood Ratio | 50.020 | 8 | .002 |
| N of Valid Cases | 1007 |  |  |
| a. 4 cells (22.2%) have expected count less than 5. The minimum expected count is .93. | | | |

**Level of education and Etiology of Trauma**

| **Crosstab** | | | | | | | | | | | | |
| --- | --- | --- | --- | --- | --- | --- | --- | --- | --- | --- | --- | --- |
|  | | | Etiologia traumei | | | | | | | | | Total |
|  |  |  | .0 | 1.0 | 1.9 | 2.0 | 3.0 | 4.0 | 5.0 | 7.0 | 8.0 |  |
| Nivel de educație | .0 | Count | 1 | 264 | 1 | 35 | 26 | 9 | 9 | 81 | 33 | 459 |
|  |  | % within Etiologia traumei | 50.0% | 45.2% | 50.0% | 41.7% | 44.8% | 25.7% | 60.0% | 52.6% | 60.0% | 46.4% |
|  | 1.0 | Count | 1 | 30 | 1 | 4 | 0 | 1 | 1 | 15 | 13 | 66 |
|  |  | % within Etiologia traumei | 50.0% | 5.1% | 50.0% | 4.8% | 0.0% | 2.9% | 6.7% | 9.7% | 23.6% | 6.7% |
|  | 2.0 | Count | 0 | 135 | 0 | 14 | 21 | 7 | 3 | 31 | 7 | 218 |
|  |  | % within Etiologia traumei | 0.0% | 23.1% | 0.0% | 16.7% | 36.2% | 20.0% | 20.0% | 20.1% | 12.7% | 22.0% |
|  | 3.0 | Count | 0 | 113 | 0 | 21 | 8 | 9 | 1 | 20 | 2 | 174 |
|  |  | % within Etiologia traumei | 0.0% | 19.3% | 0.0% | 25.0% | 13.8% | 25.7% | 6.7% | 13.0% | 3.6% | 17.6% |
|  | 4.0 | Count | 0 | 41 | 0 | 10 | 3 | 9 | 1 | 7 | 0 | 71 |
|  |  | % within Etiologia traumei | 0.0% | 7.0% | 0.0% | 11.9% | 5.2% | 25.7% | 6.7% | 4.5% | 0.0% | 7.2% |
|  | 8.0 | Count | 0 | 1 | 0 | 0 | 0 | 0 | 0 | 0 | 0 | 1 |
|  |  | % within Etiologia traumei | 0.0% | 0.2% | 0.0% | 0.0% | 0.0% | 0.0% | 0.0% | 0.0% | 0.0% | 0.1% |
| Total | | Count | 2 | 584 | 2 | 84 | 58 | 35 | 15 | 154 | 55 | 989 |
|  |  | % within Etiologia traumei | 100.0% | 100.0% | 100.0% | 100.0% | 100.0% | 100.0% | 100.0% | 100.0% | 100.0% | 100.0% |

| **Chi-Square Tests** | | | |
| --- | --- | --- | --- |
|  | Value | df | Asymp. Sig. (2-sided) |
| Pearson Chi-Square | 103.529^a^ | 40 | .005 |
| Likelihood Ratio | 93.648 | 40 | .000 |
| Linear-by-Linear Association | 14.571 | 1 | .000 |
| N of Valid Cases | 989 |  |  |
| a. 29 cells (53.7%) have expected count less than 5. The minimum expected count is .00. | | | |

**Mandibular fractures and Etiology of Trauma**

| **Crosstab** | | | | | | | | | | | | |
| --- | --- | --- | --- | --- | --- | --- | --- | --- | --- | --- | --- | --- |
|  | | | Etiologia traumei | | | | | | | | | Total |
|  |  |  | .0 | 1.0 | 1.9 | 2.0 | 3.0 | 4.0 | 5.0 | 7.0 | 8.0 |  |
| Localizare fracturi mandibulă | .0 | Count | 0 | 140 | 0 | 37 | 30 | 20 | 6 | 49 | 19 | 301 |
|  |  | % within Etiologia traumei | 0.0% | 23.6% | 0.0% | 43.5% | 50.8% | 55.6% | 40.0% | 31.0% | 33.9% | 29.9% |
|  | 1.0 | Count | 0 | 3 | 0 | 3 | 0 | 0 | 1 | 1 | 0 | 8 |
|  |  | % within Etiologia traumei | 0.0% | 0.5% | 0.0% | 3.5% | 0.0% | 0.0% | 6.7% | 0.6% | 0.0% | 0.8% |
|  | 2.0 | Count | 0 | 18 | 0 | 3 | 4 | 0 | 0 | 7 | 2 | 34 |
|  |  | % within Etiologia traumei | 0.0% | 3.0% | 0.0% | 3.5% | 6.8% | 0.0% | 0.0% | 4.4% | 3.6% | 3.4% |
|  | 3.0 | Count | 0 | 36 | 1 | 7 | 2 | 2 | 2 | 15 | 6 | 71 |
|  |  | % within Etiologia traumei | 0.0% | 6.1% | 50.0% | 8.2% | 3.4% | 5.6% | 13.3% | 9.5% | 10.7% | 7.1% |
|  | 4.0 | Count | 1 | 106 | 0 | 0 | 4 | 2 | 1 | 19 | 3 | 136 |
|  |  | % within Etiologia traumei | 50.0% | 17.8% | 0.0% | 0.0% | 6.8% | 5.6% | 6.7% | 12.0% | 5.4% | 13.5% |
|  | 5.0 | Count | 0 | 2 | 0 | 0 | 1 | 0 | 0 | 1 | 2 | 6 |
|  |  | % within Etiologia traumei | 0.0% | 0.3% | 0.0% | 0.0% | 1.7% | 0.0% | 0.0% | 0.6% | 3.6% | 0.6% |
|  | 6.0 | Count | 0 | 39 | 0 | 4 | 3 | 3 | 0 | 16 | 4 | 69 |
|  |  | % within Etiologia traumei | 0.0% | 6.6% | 0.0% | 4.7% | 5.1% | 8.3% | 0.0% | 10.1% | 7.1% | 6.9% |
|  | 8.0 | Count | 0 | 0 | 0 | 2 | 1 | 1 | 0 | 1 | 0 | 5 |
|  |  | % within Etiologia traumei | 0.0% | 0.0% | 0.0% | 2.4% | 1.7% | 2.8% | 0.0% | 0.6% | 0.0% | 0.5% |
|  | 9.0 | Count | 0 | 3 | 0 | 1 | 0 | 1 | 0 | 1 | 2 | 8 |
|  |  | % within Etiologia traumei | 0.0% | 0.5% | 0.0% | 1.2% | 0.0% | 2.8% | 0.0% | 0.6% | 3.6% | 0.8% |
|  | cominutiva | Count | 1 | 247 | 1 | 28 | 14 | 7 | 5 | 48 | 18 | 369 |
|  |  | % within Etiologia traumei | 50.0% | 41.6% | 50.0% | 32.9% | 23.7% | 19.4% | 33.3% | 30.4% | 32.1% | 36.6% |
| Total | | Count | 2 | 594 | 2 | 85 | 59 | 36 | 15 | 158 | 56 | 1007 |
|  |  | % within Etiologia traumei | 100.0% | 100.0% | 100.0% | 100.0% | 100.0% | 100.0% | 100.0% | 100.0% | 100.0% | 100.0% |

| **Chi-Square Tests** | | | |
| --- | --- | --- | --- |
|  | Value | df | Asymp. Sig. (2-sided) |
| Pearson Chi-Square | 144.374^a^ | 72 | .001 |
| Likelihood Ratio | 139.695 | 72 | .000 |
| Linear-by-Linear Association | 8.843 | 1 | .003 |
| N of Valid Cases | 1007 |  |  |
| a. 64 cells (71.1%) have expected count less than 5. The minimum expected count is .01. | | | |

**Midface location and Etiology of Trauma**

| **Crosstab** | | | | | | | | | | | | |
| --- | --- | --- | --- | --- | --- | --- | --- | --- | --- | --- | --- | --- |
|  | | | Etiologia traumei | | | | | | | | | Total |
|  |  |  | .0 | 1.0 | 1.9 | 2.0 | 3.0 | 4.0 | 5.0 | 7.0 | 8.0 |  |
| Localizare maxilar | .0 | Count | 2 | 425 | 1 | 25 | 27 | 15 | 7 | 95 | 29 | 626 |
|  |  | % within Etiologia traumei | 100.0% | 71.5% | 50.0% | 29.4% | 45.8% | 41.7% | 46.7% | 60.1% | 51.8% | 62.2% |
|  | 1.0 | Count | 0 | 0 | 0 | 0 | 1 | 0 | 0 | 2 | 1 | 4 |
|  |  | % within Etiologia traumei | 0.0% | 0.0% | 0.0% | 0.0% | 1.7% | 0.0% | 0.0% | 1.3% | 1.8% | 0.4% |
|  | 2.0 | Count | 0 | 5 | 0 | 1 | 0 | 0 | 1 | 2 | 0 | 9 |
|  |  | % within Etiologia traumei | 0.0% | 0.8% | 0.0% | 1.2% | 0.0% | 0.0% | 6.7% | 1.3% | 0.0% | 0.9% |
|  | 3.0 | Count | 0 | 1 | 0 | 1 | 2 | 0 | 1 | 0 | 0 | 5 |
|  |  | % within Etiologia traumei | 0.0% | 0.2% | 0.0% | 1.2% | 3.4% | 0.0% | 6.7% | 0.0% | 0.0% | 0.5% |
|  | 4.0 | Count | 0 | 87 | 1 | 19 | 10 | 11 | 2 | 29 | 13 | 172 |
|  |  | % within Etiologia traumei | 0.0% | 14.6% | 50.0% | 22.4% | 16.9% | 30.6% | 13.3% | 18.4% | 23.2% | 17.1% |
|  | 5.0 | Count | 0 | 30 | 0 | 6 | 4 | 7 | 0 | 10 | 0 | 57 |
|  |  | % within Etiologia traumei | 0.0% | 5.1% | 0.0% | 7.1% | 6.8% | 19.4% | 0.0% | 6.3% | 0.0% | 5.7% |
|  | 6.0 | Count | 0 | 9 | 0 | 6 | 4 | 1 | 0 | 4 | 3 | 27 |
|  |  | % within Etiologia traumei | 0.0% | 1.5% | 0.0% | 7.1% | 6.8% | 2.8% | 0.0% | 2.5% | 5.4% | 2.7% |
|  | 7.0 | Count | 0 | 0 | 0 | 1 | 2 | 1 | 0 | 1 | 0 | 5 |
|  |  | % within Etiologia traumei | 0.0% | 0.0% | 0.0% | 1.2% | 3.4% | 2.8% | 0.0% | 0.6% | 0.0% | 0.5% |
|  | 8.0 | Count | 0 | 1 | 0 | 0 | 0 | 0 | 0 | 0 | 0 | 1 |
|  |  | % within Etiologia traumei | 0.0% | 0.2% | 0.0% | 0.0% | 0.0% | 0.0% | 0.0% | 0.0% | 0.0% | 0.1% |
|  | cominutiva | Count | 0 | 36 | 0 | 26 | 9 | 1 | 4 | 15 | 10 | 101 |
|  |  | % within Etiologia traumei | 0.0% | 6.1% | 0.0% | 30.6% | 15.3% | 2.8% | 26.7% | 9.5% | 17.9% | 10.0% |
| Total | | Count | 2 | 594 | 2 | 85 | 59 | 36 | 15 | 158 | 56 | 1007 |
|  |  | % within Etiologia traumei | 100.0% | 100.0% | 100.0% | 100.0% | 100.0% | 100.0% | 100.0% | 100.0% | 100.0% | 100.0% |

| **Chi-Square Tests** | | | |
| --- | --- | --- | --- |
|  | Value | df | Asymp. Sig. (2-sided) |
| Pearson Chi-Square | 191.173^a^ | 72 | .001 |
| Likelihood Ratio | 159.916 | 72 | .000 |
| Linear-by-Linear Association | 10.827 | 1 | .001 |
| N of Valid Cases | 1007 |  |  |
| a. 68 cells (75.6%) have expected count less than 5. The minimum expected count is .00. | | | |
